# Fluidal pyroclasts reveal the intensity of peralkaline rhyolite pumice cone eruptions

**DOI:** 10.1038/s41467-019-09947-8

**Published:** 2019-05-01

**Authors:** Ben Clarke, Eliza S. Calder, Firawalin Dessalegn, Karen Fontijn, Joaquín A. Cortés, Mark Naylor, Ian Butler, William Hutchison, Gezahegn Yirgu

**Affiliations:** 1 School of GeoSciences, University of Edinburgh, Grant Institute, James Hutton Road, King’s Buildings, Edinburgh, EH9 3FE UK; 2 CNCS, Wollega University, Nekemte, Ethiopia; 3 Department of Geosciences, Environment and Society, Université Libre de Bruxelles, CP 160/02, 50, Avenue F.D. Roosevelt, B-1050 Brussels, Belgium; 4 Department of Earth Sciences, University of Oxford, 3 South Parks Road, Oxford, OX1 3AN UK; 5 Department of Geography, Edge Hill University, St. Helens Rd, Ormskirk, L39 4QP UK; 6 School of Earth and Environmental Sciences, University of St. Andrews, Irvine Building, St. Andrews, KY16 9AL UK; 7 Department of Earth Sciences, Addis Ababa University, King George VI Street, Addis Ababa, P.O. Box: 1176 Ethiopia

**Keywords:** Natural hazards, Volcanology

## Abstract

Peralkaline rhyolites are medium to low viscosity, volatile-rich magmas typically associated with rift zones and extensional settings. The dynamics of peralkaline rhyolite eruptions remain elusive with no direct observations recorded, significantly hindering the assessment of hazard and risk. Here we describe uniquely-preserved, fluidal-shaped pyroclasts found within pumice cone deposits at Aluto, a peralkaline rhyolite caldera in the Main Ethiopian Rift. We use a combination of field-observations, geochemistry, X-ray computed microtomography (XCT) and thermal-modelling to investigate how these pyroclasts are formed. We find that they deform during flight and, depending on size, quench prior to deposition or continue to inflate then quench in-situ. These findings reveal important characteristics of the eruptions that gave rise to them: that despite the relatively low viscosity of these magmas, and similarities to basaltic scoria-cone deposits, moderate to intense, unstable, eruption columns are developed; meaning that such eruptions can generate extensive tephra-fall and pyroclastic density currents.

## Abstract (web summary)

Dynamics of peralkaline rhyolite eruptions remain elusive due to the lack of direct observations. Here the authors provide X-ray Computed Tomography, thermal modelling and field data of fluidal shaped pyroclasts and show that peralkaline rhyolite pumice cones are the product of moderate to intense eruptions.

## Introduction

Peralkaline rhyolites (molar NaO_2_ + K_2_O/Al_2_O_3_ > 1) are medium to low-viscosity volatile rich magmas and are found in rift zones worldwide^1^. Despite this, relatively little is known about the style and eruption dynamics of volcanism characterised by them. Eruption dynamics are typically controlled by the interplay between the geochemical and rheological factors influencing the effectiveness of degassing. Peralkaline rhyolite melts exhibit low viscosities compared to other rhyolites of equivalent silica enrichment, a property attributed to the depolymerising effects of excess alkalis and high halogen concentrations^2–5^. Peralkaline rhyolite eruptions are commonly associated with an enigmatic suite of eruption products including, but not limited to, obsidian lava flows, welded ignimbrites and pumice cones. Overall, the absence of direct observations of peralkaline eruptions, the unusual geochemistry of the melts, and the fact that deposits from peralkaline rhyolite eruptions do not fall neatly into established eruption classifications means that the eruption styles of peralkaline rhyolites are largely unknown. The evaluation of hazard and risk at a given volcano is contingent on having a robust conceptual model of how the volcanic system operates and the nature of its associated eruptive activity. For peralkaline volcanoes worldwide, the paucity of this knowledge is a significant limitation.

The East African Rift (EAR) is characterised by the greatest density of silicic peralkaline volcanoes on Earth, and furthermore, Ethiopia is regarded as the fifth most volcanically threatened country^6^; with 10 million people living within 30 km of a volcano^7^. Here, we present a study of unusual, yet uniquely well preserved, fluidal-shaped pyroclasts found in peralkaline rhyolite pumice-cone deposits at Aluto volcano in the Main Ethiopian Rift (MER) (a northerly segment of the EAR). Somewhat similar pyroclasts, termed globules, have been observed in peralkaline rhyolite deposits on Gran Canaria, Pantelleria and in the Kenyan rift^8–10^, and are characterised as fluidly shaped glassy pyroclasts with a thin skin and highly vesicular interior. There is currently no consensus on how they form or what style of eruptive activity they are indicative of. Here, for the first time, these pyroclasts are investigated in detail using scanning electron microscopy (SEM) and X-ray computed microtomography (XCT) providing unique insights into their 3D structure. We present field and clast observations, undertake thermal modelling and develop the first comprehensive conceptual model for the formation of these peralkaline fluidal-shaped clasts, henceforth referred to as pumiceous achneliths. This new understanding of pumiceous achneliths forms the basis of a conceptual model of pumice-cone forming peralkaline rhyolite eruptions and provides some important constraints on this hitherto ambiguous style of activity. This allows for a better understanding of the likely hazards associated with future eruptions of peralkaline rhyolite volcanoes, potentially worldwide.

## Results

### Aluto caldera

**Fig. 1:**
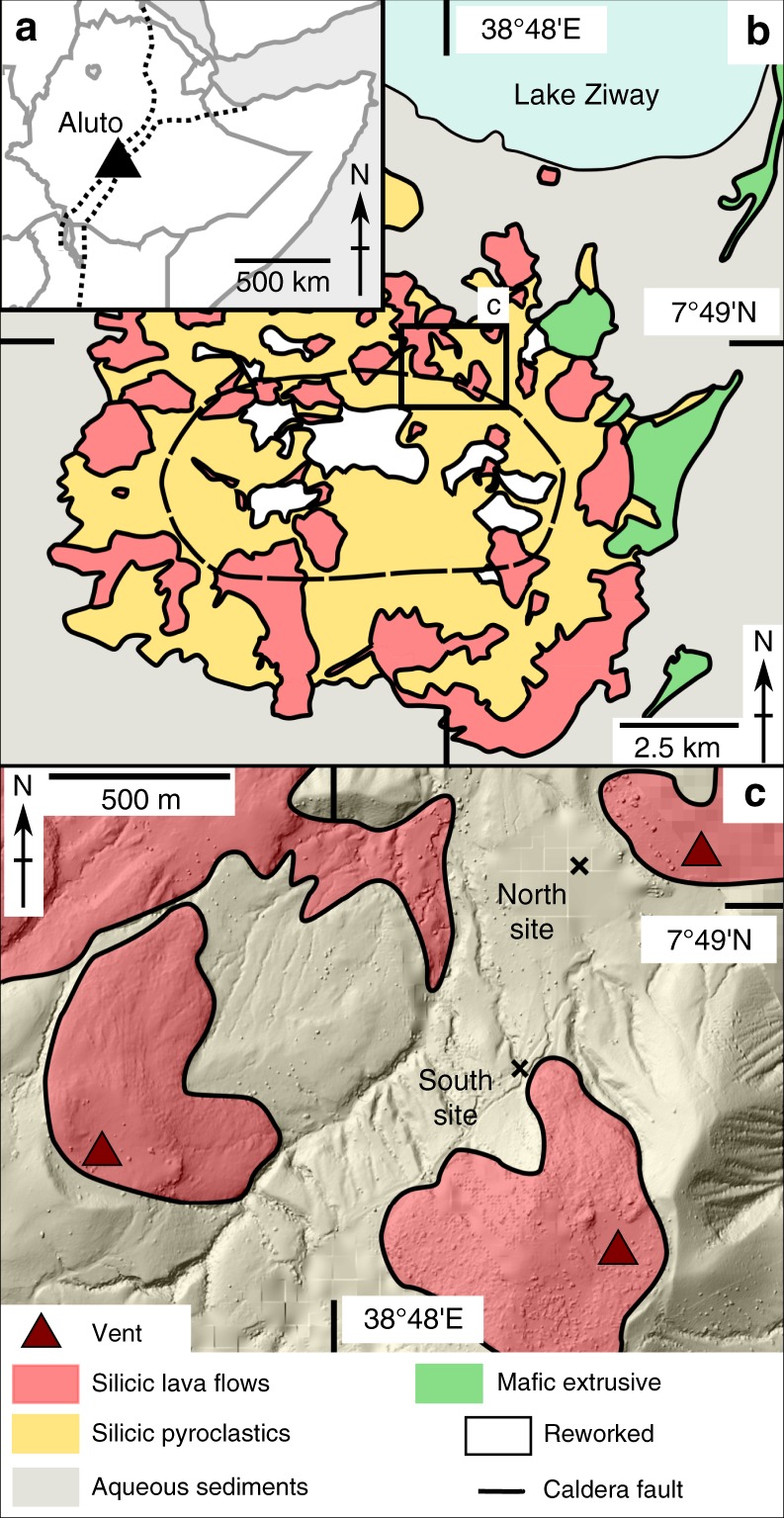
Contextual and simplified geological map of Aluto caldera and the sampling sites. **a** Map showing location of Aluto in Ethiopia. **b** Simplified geological map of Aluto after Hutchison, 2015. **c** DEM hillshade overlain by geology showing north and south study sites. DEM produced using Aluto LiDAR data from ref. ^11^

Aluto is a 12 km wide caldera complex in the central MER situated directly south of Lake Ziway (Fig. 1)^11^. The complex underwent major explosive eruptions at around 310 ka, likely associated with caldera collapse, and since at least ~60 ka it has erupted a series of smaller scale pyroclastic deposits and obsidian lava flows across the main edifice^11,12^. This recent volcanism (<60 ka) is dominated by peralkaline rhyolite magmas, thought to be the product of protracted (>80%) fractional crystallisation of a basaltic parental melt^13^. At Aluto, these eruptions typically entail the formation of a pumice cone with, in most cases, pyroclastic density current (PDC) deposits followed by the emplacement of obsidian lava flows. Pumice cones are a volcanic landform commonly produced during peralkaline rhyolite eruptions, and due to their resemblance to basaltic scoria cones, have generally been considered to develop during relatively low-intensity strombolian-style eruptions^14,15^. The presence of PDC deposits from pumice cones at Aluto and elsewhere (i.e., Monte Pilato, Lipari^16^) suggests this assumption may not be universally correct.

### Deposit description

The pumiceous achneliths on which this study is based are sourced from two separate sites (termed here north and south site, respectively) within pumice cone deposits adjacent to, and stratigraphically below, obsidian lava flows in the NE section of the caldera (Fig. 1). The north and south sites are 600 m apart (Fig. 1), but are proximal to, and stratigraphically below different obsidian lava flows. They are also stratigraphically discontinuous and so represent separate deposits. However, they are otherwise nearly identical in their lithological, sedimentological, and chemical characteristics. The deposits are each 2–3 m thick and comprise unconsolidated, poorly sorted massive breccias. They are massive, clast supported, and ash and matrix poor.

The North and South deposits represent the proximal deposits of pumice cones (estimated 300 and 600 m from the likely source vents respectively, Fig. 1) produced prior to the emplacement of their associated obsidian lava flows. To account for their very poor sorting yet high angularity, these deposits either represent proximal fall deposits produced during a prolonged but highly unsteady eruption, as seen in violent strombolian eruptions^17^, or fall deposits which have been very-locally remobilized (avalanched), from their parent cone.

### Clast descriptions

**Fig. 2:**
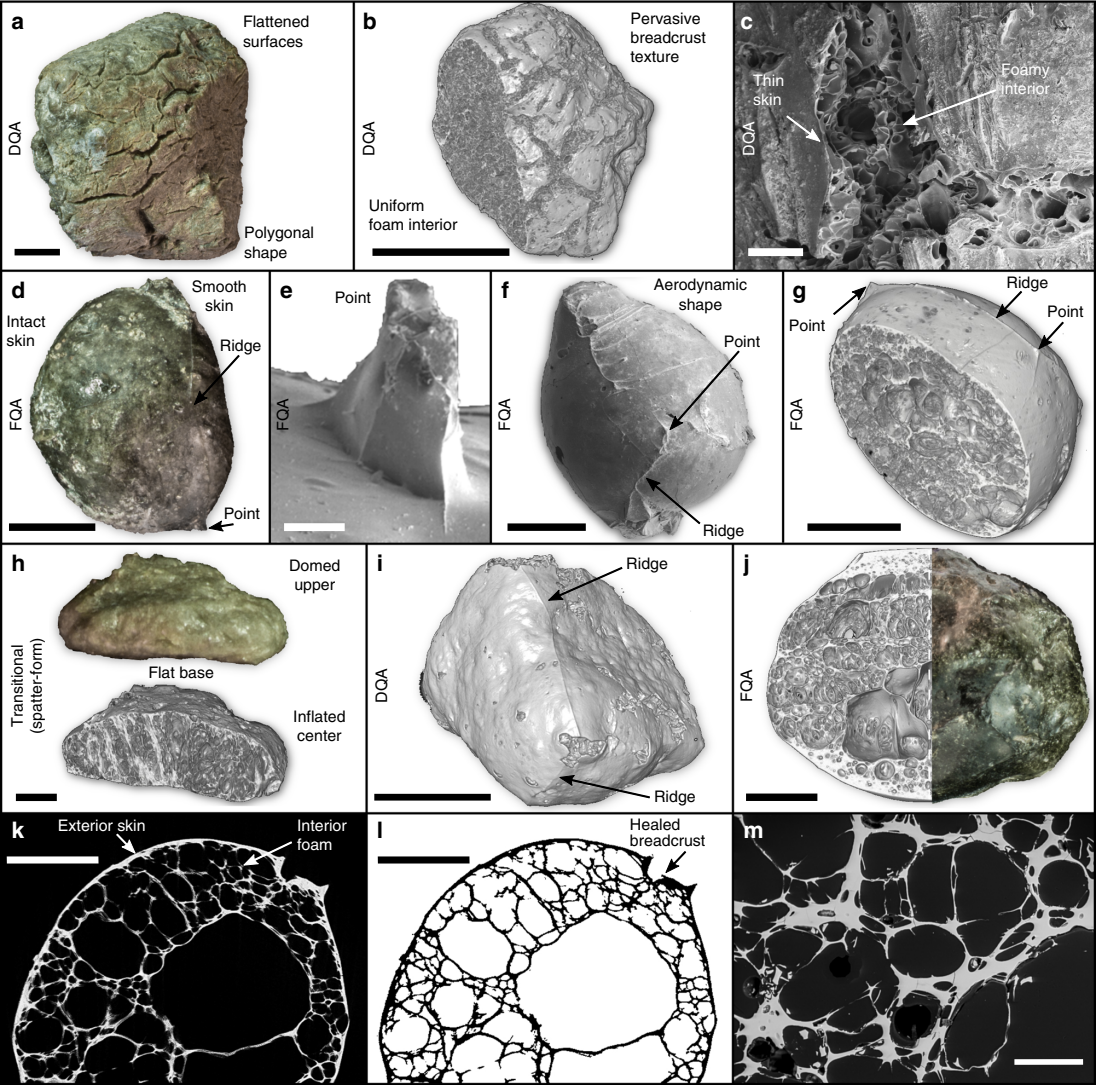
Images displaying the primary features of pumiceous achneliths. **a** Photograph of a deposit quenched achnelith (DQA) (scalebar = 5 mm). **b** Seconday electron scanning electron microscope (SE SEM) image of a DQA (scalebar = 5 mm); **c** 3D rendering of an X-ray microtomographic (XCT) scan showing a DQA (scalebar = 200 μm). **d** Photograph of a flight quenched achnelith (FQA) (scalebar = 5 mm), **e** SE SEM image of a point structure on an FQA (scalebar = 200 μm); **f** SE SEM image of an FQA; **g** 3D rendering of XCT of an FQA (scalebar =1 mm); **h** Photograph and 3D rendering of spatter-form lapillus, displaying aspects of FQA and DQA origins (i.e., deformed droplet) (scalebar = 1 mm); **i** 3D rendering of DQA showing remnant ridge structures identical to those seen on flight quenched achneliths (scalebar = 5 mm); **j** photograph and 3D rendering of a spherical FQA showing bands of variable vesicle size, shape, and number density (scalebar = 5 mm). **k** Reconstructed XCT slice showing a cross-section through an FQA, displaying the basic structure of internal foam with a thin exterior skin (scalebar = 1 mm). **l** Binarized XCT slice (same as **k**) showing a healed breadcrust crack. This also exemplifies the data used for bulk clast volume calculation (scalebar = 1 mm). **m** Electron backscatter image showing the glass foam network within a DQA. Larger vesicles have retracted internal bubble walls indicative of bubble coalescence (scalebar = 200 μm)

**Fig. 3:**
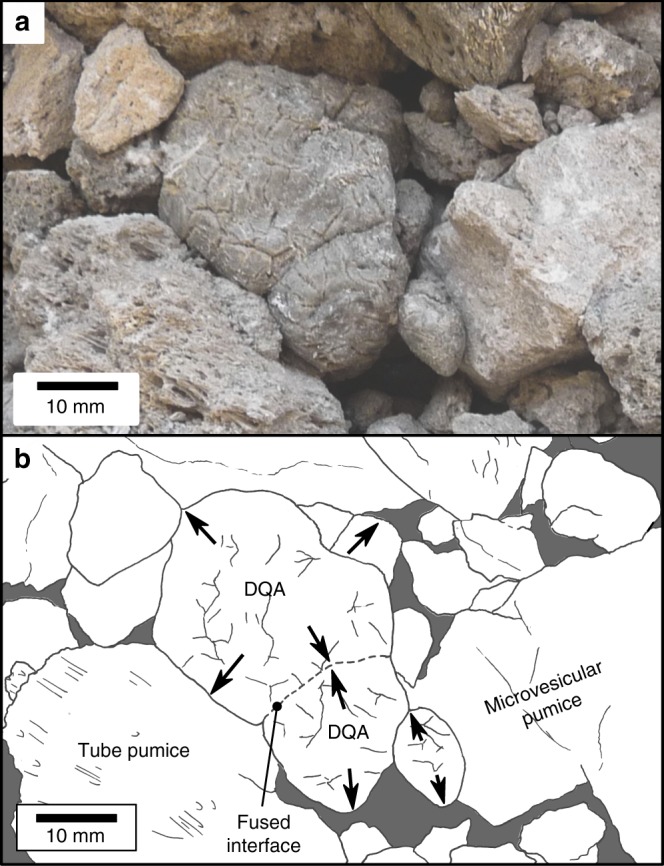
Images showing the in-situ inflation of deposit quenched achneliths. **a** Image of deposit quenched achneliths, showing evidence of in situ inflation into void spaces between and against adjacent pumices. Note the polygonal form of the achneltih as a result of compression against adjacent clasts. **b** Annotated and line drawing of the same image. Arrows indicate the inflation of the DQAs. The central DQA is formed by the fusion of two adjacent DQAs, forming a single pyroclast

The deposits contain clasts of tube pumice, microvesicular pumice (total pumice: 80 wt%), dense obsidian bread crust bombs (5 wt%), assorted siliciclastic and ignimbrite lithics (5 wt%), and pumiceous achneliths (10 wt%). All clasts other than the pumiceous achneliths are angular. The achneliths are a very distinctive clast type; they are well- to sub-rounded in form, have a thin (10–100 μm) smooth grey-to-black glassy skin and a highly vesicular interior (Fig. 2). In situ, pumiceous achneliths have highly irregular polygonal shapes, fill their void space in the deposit, and press-up and deform against adjacent clasts (Fig. 3). They also span a range of sizes, from mm to cm (to decimetres at other localities). There is no systematic spatial distribution of clast types throughout the deposit.

In thin section, some pumices contain small (<20 μm) microlites but the pumiceous achneliths are aphyric. EPMA glass analyses (see Supplementary Data 1) show that all juvenile clasts are peralkaline rhyolites (pantellerites) with a mean glass SiO_2_ concentration of 73 wt%, a peralkalinity index (molar Na_2_O + K_2_O/Al_2_O_3_) of 1.6, FeO_(*t*)_ of 5.4 wt%, and average F and Cl concentrations of 3700 and 1900 ppm, respectively. Different clast types are indistinguishable based on their major element geochemistry and Cl concentrations. Fluorine provides some distinction between clast types, with tube pumice having the highest concentration, microvesicular pumice and dense obsidian bombs having the least, and deposit quenched achneliths with a concentration spanning much of the range of the other clast types (see Supplementary Figs. 1–4).

The pumiceous achneliths at Aluto comprise a glassy foam body surrounded by a thin (10–100 μm) skin of smooth, black obsidian (Fig. 2). In thin section, the glasses are isotropic with no apparent alteration, suggesting they are relatively fresh and similar to their original form. Their high vesicularity (67–93 vol%) imparts a low-bulk density of 0.2–0.8 g cm^−3^. Due to resolution constraints of the XCT scans, vesicle shape can only be described qualitatively, and varies from near spherical to highly tortuous within the same clast. Many vesicles are coalesced.

**Fig. 4:**
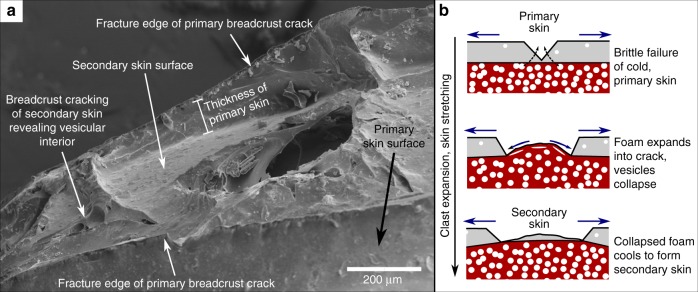
The healing process of breadcrust textures in pumiceous achneltihs. **a** Annotated oblique SEM image of a breadcrust scar on an FQA. **b** Conceptual model showing the formation of breadcrust scars, a feature developed during the transition between brittle and ductile skin regimes in the growth of pumiceous achneliths. This process enhances cooling of the internal foam, and keeps the surface temperature higher allowing extended ductile behaviour by advection

The pumiceous achneliths can be subdivided into two morphologically distinct groups: flight-quenched achneliths (FQAs), distinguished because they possess aerodynamic, spherical to teardrop shapes (Fig. 2d–g, j) and deposit-quenched achneliths (DQAs), distinguished because of their highly irregular, polygonal shapes formed through in situ inflation (Figs. 2a, b, i and 3). In both groups, the delicate outer obsidian skin is still intact (Fig. 2d, f, g, i, k, l) and confirms clast roundness is not imparted by abrasion during transport. FQAs tend to be smaller than DQAs, and in these deposits have mean diameters (of their equivalent sphere) of 4 and 15.5 mm, respectively. However, DQAs up to 30 cm in length have been found elsewhere at Aluto. There is no clear difference in vesicularity, nor vesicle shape between FQAs and DQAs. It is important to note that breadcrust textures are present in both FQAs and DQAs. However, DQAs display wider cracks, which are better connected, and are present more frequently than in FQAs. In some examples, breadcrust cracks have apparently healed; where ductile melt from the interior has reformed a skin in the place of the crack (Figs. 2k, l and 4). FQAs often display a surficial point-and-ridge structure, with points in a roughly tetrahedral coordination around the teardrop-shaped to sub-spherical clast joined on the surface by small ridges (Fig. 2d–f, g). A remnant version of this structure can often be seen on DQAs (Fig. 2i).

There are two end member textures of pumice in the deposits: tube pumice, with very high vesicle aspect ratios and microvesicular pumice, which has highly tortuous microvesicles that lack any preferred orientation and have a small aspect ratio. Pumice is sometimes heterogenous on a centimetre scale, containing zones of both pumice texture end members. Dense, angular obsidian bread crust bombs are also present. They possess macrovesicular cores and are significantly denser than both the pumiceous achneliths and the pumice. It is not clear whether these clasts are juvenile or accessory. Conduit-derived, accessory lithic components represent pre-Aluto as well as Aluto stratigraphy: with small (2.5 mm mean diameter), oxidised, angular fragments of siliciclastic sediments, and green welded ignimbrite (the ‘Qgei’ ignimbrite of ref. ^11^).

### Thermal modelling

**Fig. 5:**
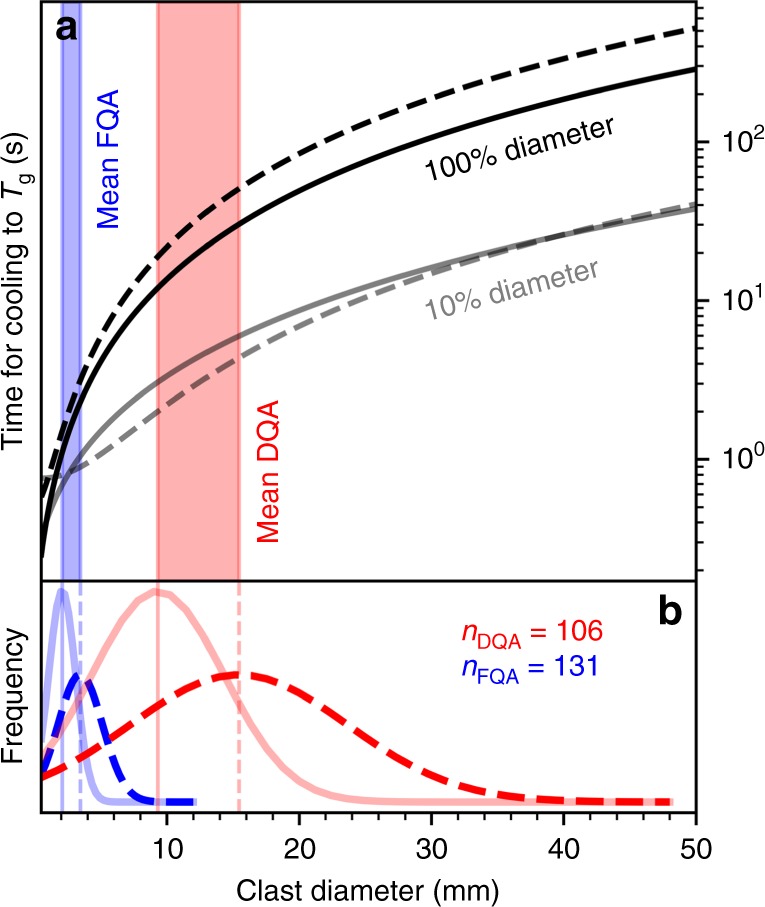
The cooling rates of pumiceous achneliths during flight by clast diameter. **a** The results of the thermal modelling with the black and grey lines representing the time taken for 100% and 10% of the diameters, respectively to cool below *T*_g_. The dashed lines show the results assuming a vesicularity of 0.9, whilst the solid lines represent a vesicularity of 0, thus bracketing the true vesicularity for any given pyroclast. **b** Best-fit gaussian size distributions of FQAs (blue) and DQAs (red). The dashed lines indicate the diameters of the achneliths as measured (i.e., vesicular), and the solid lines indicate the recalculated diameters once the vesicularity (assumed to be 0.9) has been removed. In both panels, the mean diameters of the original FQAs and DQAs (i.e., zero vesicularity) and the final inflated FQAs and DQAs (i.e., porosity = 0.9) are shown by vertical lines and provide the range of diameters over which the mean FQA and DQA evolved from fragmentation to quenching

The pumiceous achneliths, i.e., fluidal-shaped clasts, are unique because they represent ductile processes in a deposit of otherwise brittlely fragmented pyroclasts. To investigate whether peralkaline rhyolite magmas could remain ductile during flight, a numerical, forced-convection and conduction-based thermal model was developed (Supplementary Code 1). The time taken for different sized pyroclasts to cool to the point where 10 and 100% of the pyroclast’s radius was below the estimated glass transition temperature (*T*_g_) was determined. It is important to note that here we define *T*_g_ to be the temperature at which the melt with 1 wt% H_2_O reached 10^12^ Pa s: 400 °C according to the model of ref. ^3^. The results of the thermal model are presented in Fig. 5. Taking the mean clast diameter for DQA clasts from the clast-size histogram we show that the whole clast (i.e., 100% diameter) will have cooled to below *T*_g_ after 15–50 s. In contrast, a FQA of mean diameter will have reached the same point by only 2 s. For comparison, cooling of the outermost 10% (by diameter) is in the order of a few seconds for both clast types. We repeated the model runs using a range of possible *T*_g_s, the results suggest that cooling times are relatively insensitive to this parameter within a realistic range of input values. For example, where *T*_g_ = 520 °C, the point at which a peralkaline rhyolite with 0.1 wt% H_2_O should have a viscosity of 10^12^ Pa s^3^, the resulting difference in the cooling time for exteriors of the mean FQA and DQA are within model precision, and for the interior of the mean DQA (where the difference should be greatest) is of the order of 5 s.

## Discussion

Although not common, clasts similar to our pumiceous achneliths, previously referred to as globules, have been observed within welded ignimrbites elsewhere. Globules have not been formally defined but are described as comprising either a foam or single central vesicle surrounded by a thin skin. These globules have be found within welded ignimbrites at Mt. Suswa (Kenya)^10,18^, Pantelleria, and Gran Canaria^8^. In each of these cases, the ignimbrites are peralkaline trachyte in composition. The Monte Pilato pumice cone on Lipari contains some clasts that closely resemble DQAs, but are simply considered pumice by the authors (Fig. 5e in ref. ^16^). Those clasts are found in unconsolidated fall and flow deposits and are high-K rhyolites. As seen at Aluto, and in all the above cases there is evidence for post-deposition expansion of the pyroclasts. There is currently no consensus on the origin of globules; some authors suggest they represent reheated, brittlely fragmented shards reshaped during flight^18^, whilst others invoke ambiguous, non-brittle fragmentation in eruptions that are dynamically similar to basaltic fire fountaining, yet still produce PDCs^19^. A common feature of the globules described previously, and their proposed origins, is the shaping of the clasts by surface tension, defining them as achneliths; a form of pyroclast first described in basaltic systems^20^. To form an achnelith, the structural relaxation timescale (*τ*) of the melt (i.e., the time required for stress to be relaxed by ductile, structural reorganisation of the melt) must be less than the timescale for cooling to the glass transition (*t*_tg_) during flight. This (i.e., *τ* < *t*_tg_) allows for ductile, surface tension driven deformation^21^.

**Fig. 6:**
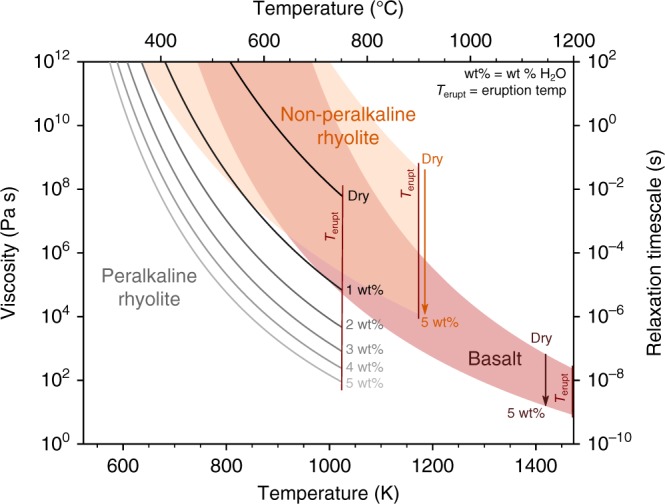
Viscosity-temperature relationship for peralkaline rhyolite melt with varying water concentration. Pantellerites typically contain between 2.5 and 5 wt% H_2_O prior to eruptive degassing^27,28^. The melt viscosity for pantellerites is calculated using the model of ref. ^3^. For comparison, the viscosity-temperature relationship calculated by the VFT equation^23^ is shown for a typical, non-peralkaline (in this case peraluminous), rhyolitic melt (Rhyolite Glass Mountain – 1 standard) and basaltic melt (Basalt Hawaii Volcanic Observatory – 1 standard). Note that at eruption temperature, hydrous Aluto peralkaline rhyolites have a similar melt viscosity to basalts, and possess exceptionally low glass transition temperatures (temperature at which viscosity = 10^12^ Pa s)

The structural relaxation time of a given melt is a function of its viscosity and the bulk shear modulus (*τ* = *η*/*G*)^22^. The viscosity (*η*) of the melts that form pumiceous achneliths at Aluto has been estimated using the VFT equation^23^ with modified fit parameters for peralkaline rhyolites^3^ (Fig. 6). It should be noted that F has a network modifying influence on the viscosity of silicate melts^24^ of the same magnitude as water on a wt% basis^25^, and the peralkaline rhyolite viscosity model^3^ does not explicitly take F into account in model coefficients. However, F concentrations in the samples used to calibrate the model (pumice from the same eruption: mean 3900 ppm F^26^) are very similar to those measured in glasses at Aluto (mean 3700 ppm F). The model is therefore considered to implicitly take the influence of F into account for the Aluto samples.

The aphyric nature of the pumiceous achneliths at Aluto precludes any standard geothermometry. Instead, we assume eruption temperatures around 750 °C, the storage temperature of other pantellerites from Aluto, based on the alkali-feldspar—melt geothermometer^13^. At these temperatures, we estimate the viscosity of the melts when dry to be around 10^7.7^ Pa s. However, pantellerites typically contain between 2.5 and 5 wt% H_2_O prior to eruptive degassing^27,28^, and at their eruption temperature have melt viscosities similar to a basalt^3,23,29^ (Fig. 6). The presence of just 1 wt% H_2_O, for example, reduces the viscosity at 750 °C to just 10^4.9^ Pa s. At this viscosity, *τ* = 10^−5.1^ s. Cooling times (*t*_tg_) are significantly longer than this (0.7–50 s) for pumiceous achneliths (Fig. 5), confirming *τ* < *t*_tg_, and that reshaping by surface tension is possible. The existence of achneliths in pyroclastic peralkaline rhyolite deposits therefore provides an important real-world validation of the inferred low viscosity and glass transition temperatures of these magmas previously determined experimentally^3–5^.

**Fig. 7:**
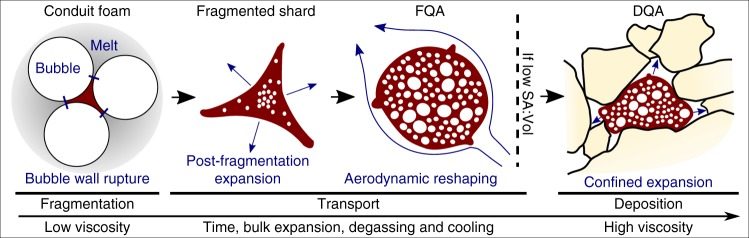
Conceptual model showing the generation of flight quenched achneliths (FQAs) and deposit quenched achneliths (DQAs). A foam is first fragmented generating shards, due to their low viscosity, they continue to degas. As the skin forms through cooling, they become a closed system and inflate. Inflation continues through transport and the clasts are shaped aerodynamically and to minimise free surface energy. If the surface area to volume ratio (SA:Vol) is high, they harden before impact forming FQAs. If the SA:Vol is low, they remain plastic post-deposition and expand into place forming DQAs

The sequence of mechanisms by which pumiceous achneliths are formed can, to an extent, be deciphered from their texture and surface features. Typical spheroidal achneliths, such as those formed in Hawaiian fire-fountain eruptions (e.g., Pele’s tears) are thought to be produced during non-brittle fragmentation of a low-viscosity basalt magma^20,21,30^. However, we infer primary fragmentation for the pumiceous achneliths to have been brittle. This is evidenced by the point-and-ridge structures on the surface of many achneliths (both DQAs and FQAs) (Fig. 2e–g, i) which we propose are retracted bubble walls between fragments of bubbly conduit magma (Fig. 7). The smaller achneliths originate as cuspate shards whilst the larger achneliths may originate as clots of bubbly magma. Post-fragmentation cooling and degassing at the clast surface develops a coherent skin of higher viscosity melt, and the associated reduction in volatile diffusion rates prevents significant gas flux from the fragments. The fragments thus form partially closed systems and inflate during progressive degassing and flight. It is possible that basaltic achneliths form in a similar manner, only their prolonged fluidal behaviour and lower viscosities mean that such surface features may have been lost.

The aerodynamic morphologies of the pumiceous achneliths at Aluto show that they were still capable of ductile deformation after apparently brittle fragmentation. For such low viscosity melts to fragment requires rapid decompressive stresses, such that strain cannot be structurally relaxed. The fragmentation styles of pantelleritic pumices from the Cuddia di Mida pumice cone eruption were investigated, and it was found that the required conduit-averaged decompressive strain rates implied unrealistically high mass fluxes (>10^8^ kg s^−1^) (ref. ^31^). A potential solution is to invoke strain localisation, where spatial variability in water content, microlite content, bubble number density and size can generate high-strain-rate shear zones, where the strain rate is sufficiently magnified to elicit brittle fragmentation^32^. At Aluto, there is clear cross-conduit variability in the rheological behaviour of magma; where some fragments produce aphyric fluidal achneliths, while the remainder form microlite-bearing brittlely fragmented, angular pumice. The pumices also display variability in bubble elongation and bubble number density (coevally produced tube pumice and microvesicular pumice), which shows that strain conditions vary across the conduit^33^, and further supports the notion that strain localisation may have played a role in the brittle fragmentation of low-viscosity magma at Aluto^32,34^.

The process of inflation is elucidated by textural variations and the presence of both breadcrusted and non-breadcrusted pumiceous achneliths (Fig. 2). This shows that inflation occurs in two end-member regimes: this first being ductile skin behaviour, where the rate of strain experienced by the skin due to wholesale clast expansion (aka. rate of skin stretching) does not exceed the rate at which it can be structurally relaxed $\left( {\frac{1}{\tau }} \right)$ and so expansion is accommodated ductilely. The second regime results in brittle skin behaviour, where strain accumulates at a rate greater than it can be structurally relaxed, resulting in brittle cracking (bread-crusting). The transition from the ductile to brittle skin regime occurs where the strain rate exceeds the structural relaxation rate of the outer portion of the pyroclast. The rate of stretching of the outer portion of the pyroclast is a function of the internal bubble growth rate. The rate of internal bubble growth is liable to be greater at lower viscosities^35^, and so reduces with continued degassing and cooling. When in the brittle skin regime, the interior must be sufficiently low viscosity to allow bubble growth to induce strain in the outer portions of the pyroclast, but the outer portion of the clast must be sufficiently viscous for the strain rate to induce a brittle response. There is a delicate balance between cooling, degassing, and bubble growth rates, but the balance is tipped towards brittle skin behaviour in larger pyroclasts, where the interior remains thermally insulated and low viscosity, whilst the outer is cold and brittle. In smaller pyroclasts, the interior is less insulated, and so cools at a similar rate to the skin; undergoing wholesale quenching before entering the brittle skin regime. This explains why larger bread crust textures develop more often in the larger DQAs than in the smaller FQAs. This also suggests that for small FQAs, expansion must occur rapidly and early-on in the ductile skin regime, before the skin has quenched (in under 2 s (FQA curves in Fig. 5)).

The skin of the pumiceous achneliths is particularly thin (10–100 μm) compared to typical millimetre scale of breadcrust bomb rinds^36,37^. This likely results from expansion during regime 1, where the ductile skin is stretched to cover the larger surface area and is necessarily thinned. In some breadcrusted FQAs there is evidence of transitional behaviour between the end-member ductile and brittle skin regimes: where breadcrust cracks have healed (Figs. 2k, l and 4). Here, we propose that the skin has become sufficiently viscous to crack brittlely, exposing the bubble rich interior through continued expansion. The hotter interior behaves in a ductile fashion, so vesicles collapse by diffusive degassing, bubble burst, and stretching. This process forms a new coherent skin, regenerating the surface, and replacing the crack with a scar (Fig. 4). This translation of hot material under the skin to the surface advects heat, acting both to maintain ductility at the surface of the clast, allowing reshaping, whilst also cooling the interior. During the ductile skin regime, surface tension and drag reshapes the clasts to attain their spherical to droplet shape. As the free surface energy is directly proportional to the surface area, and the surface area is proportionally smaller at larger volumes, shaping by surface tension is liable to be more pronounced in smaller clast diameters, which is shown by the relative shapes of DQAs and FQAs, and also recognised in basaltic achneliths^21^. Though surface tension should act to remove irregularities such as the surficial point-and-ridge structures, the high surface area of the points and ridges will promote rapid cooling, increasing their viscosity and preserving their shape whilst the main body of the clast continues to inflate and reshape.

The existence of FQAs and DQAs indicates there is significant variability in the rheology of different pumiceous achneliths throughout deposition. The aerodynamic shapes and relative paucity of bread crust textures in FQAs (Fig. 2) implies that they cooled below their glass transition temperature in-flight, quenched before entering the brittle skin regime, and were not deformable upon impact. The DQAs, however, remain above, or exceed their glass transition temperature until after deposition to allow for inflation and plastic deformation against adjacent clasts in the deposit (Fig. 7). We propose that although these clasts represent the same eruptive material, their respective sizes result in distinct temperature-time paths after fragmentation. The small size and higher surface area to volume ratio of FQAs resulted in a faster cooling rate, allowing them to embrittle before impact. The larger DQAs retained heat longer, remaining plastic post-deposition, allowing vesiculation and expansion to continue. This is confirmed by the results of the thermal model, that demonstrate in most cases, the disparity of behaviour between FQAs and DQAs can be explained by their relative sizes: the mean FQA has completely cooled by around 3 s, whereas this takes between 15 and 50 s for the mean DQA (Fig. 5). FQAs are therefore frozen during transit before deposition, whereas the interior of the DQAs remain above *T*_g_ and continue to degas and expand after emplacement. However, in reality there is some overlap in the diameters of FQAs and DQAs (Fig. 5), as well as transitional spatter-form clasts (Fig. 2h) which were clearly small and droplet shaped during flight but deformed on impact. This can be explained by natural variability in parameters such as initial temperature, volatile content, flight path, and the thermal influence of surrounding pyroclasts during transport. Further, the surface temperature of pyroclasts may increase after deposition; the reduced surface heat flux due to the absence of forced convection, and the persistence of conductive heat flux from the centre can allow the surface temperature to rise^38^. It may be the case therefore, that the outer portions of some pumiceous achneliths cool through their glass transition before impact but rise above it again after deposition, allowing for prolonged plastic behaviour. The post-deposition inflation of pumiceous achneliths, and partial fusion with adjacent clasts provides a degree of cohesion to these deposits to the extent where they can be considered incipiently welded. Had the deposits contained a greater proportion of pumiceous achneliths or had inflation progressed further, it is feasible that the deposit would have welded. We also note that most globules that have been found elsewhere occur within welded deposits. We therefore propose that inflation of these pyroclasts after deposition may be a significant factor contributing to the very commonly welded nature of peralkaline rhyolite pyroclastic deposits.

The time-scale of the formation of these achneliths provides important constraints on the eruption styles that generate pumice cones. Significantly, the output of the thermal model provides an estimate for the time interval between fragmentation and deposition for DQAs. As we have established, DQAs must at least in part exceed their glass transition temperature after deposition in order to deform plastically, whilst the presence of a bread-crusted skin shows that at least the outer-most portion of the DQA cooled below *T*_g_. For a DQA of any given size therefore, the time-of-flight should be somewhere between the 10% (cooled crust) and 100% (cooled interior) curves presented in Fig. 5. Here, we see that for a mean DQA from Aluto, the time between ejection from the vent and deposition was between 4 and 15–50 s. This provides an important insight on the depositional mechanism of pumice cone deposits. The cooling rates of DQAs show either that deposition occurred over a short time scale (between 4 and 50 s), or that clasts were entrained in a hot fluid prior to deposition thus minimising the cooling rate. Three reasonable scenarios that satisfy these conditions are deposition from ballistic trajectories, deposition from the turbulent edge of the lower portions of an ascending column, or entrainment in, and deposition from, a hot PDC. For these deposits, considering the high angularity of pumice clasts, and the lack of matrix, ballistic or column-edge deposition are considered most likely. In the case of the north and south deposits, DQAs have been found at distances of 300 and 600 m from their source vents. Taking the average DQA at 15.4 mm with a density of 600 kg m^−3^, and even assuming extreme conditions of ballistic transport (ejection velocity 300 m/s, 45° ejection angle and a tailwind of 20 m/s), the maximum distance such a pyroclast should reach is 185 m^39^. In addition, DQAs at Aluto are typically smaller than 10 cm in diameter, considered to be the lower limit for pyroclasts to follow ballistic trajectories^40^. For these reasons, the DQAs at Aluto are thought to be sourced from the edge of an ascending column, the flaring or inclination of which allows for the deposition of particles beyond their ballistic limit^41^, whilst minimising the duration between primary fragmentation and deposition. It should be noted however, that scenarios 1–3 are all plausible mechanisms for the production of DQAs elsewhere.

We conclude that these unusual pyroclasts—pumiceous achneliths—found in pumice cone deposits (potentially globally) provide important insights on the nature of these hitherto enigmatic eruptions: Despite their fluidal appearance and apparent low viscosity, pumiceous achneliths are initially fragmented brittlely alongside more typical pumice. Their prolonged relaxation times and degassing allow them to form their final, conspicuous morphology. We also find pumiceous achneliths are in this case not ballistically emplaced, but fall from the edge of ascending eruption columns. There is also significant conduit-wide variation in strain-rate and magma rheology to explain the presence of pumice and pumiceous achneliths within the same deposit and the brittle fragmentation of low viscosity melts. Taken together, these conclusions show that pumice cones can generate intense eruption columns. This is in contrast with prior strombolian interpretations of pumice cone eruptions, established in response to the resemblance of pumice cone structures to basaltic scoria cones^14,15^. This has important implications for the hazards posed by such eruptions: in particular, in formulating more accurate conceptual models for the associated activity. It is now clear that pumice cone eruptions may be associated with wide-spread tephra fallout, column-collapse-type PDCs, occasional cone-avalanches, and in latter eruption stages, the emplacement of obsidian lava flows. The evidence for cross-conduit variations in viscosity and strain-rate during the explosive phase of these eruptions suggests a highly unsteady and non-uniform setting. This implies that the eruptions themselves may be complex, with the potential for multiple stages of eruption column generation and collapse. Notable and somewhat similar eruptions have occurred at Chaitén (2008)^42^ and Cordón Caulle (2011)^43^; both rhyolitic with tephra fallout, PDCs and obsidian flows, but where pumice cones themselves haven’t been well documented. This provides a clear rationale for considering small to moderate PDCs as potential proximal to medial hazards around the edifices, and that moderate tephra-fall hazards (equivalent to eruptions of VEI 3–4) should be considered. In summary, pumice cone eruptions remain little studied, but are liable to be associated with more hazardous eruptions than previously assumed.

## Methods

### Bulk deposit analysis

The samples were collected during a 2-week trip to Aluto volcano, Ethiopia. The two deposits under investigation (north site and south site) were logged, photographed and a bulk sample, free of surface wash, was collected at each. Bulk samples were dried at 100 °C for at least 24 h, and sieved by hand into 1 phi size fractions and weighed. Each sieved fraction, depending on the number of clasts present, was then representatively split for manual analysis of the components.

### Deposit componentry

Clasts within each sub-sample were then assigned to 1 of 6 component types: tube pumice, microvesicular pumice, FQA, deposit-quenched achneliths (DQA), dense obsidian or lithic. These classes were then weighed and the number of clasts in each category counted. All 189 FQAs and DQAs were individually measured on three axes using a digital caliper to a precision of ±0.02 mm. As >95% of clasts were equant according to the Zingg classification^44^, the mean of the three axial measurements was considered the diameter of the pyroclast.

### Geochemistry

The glass composition of a sub-sample of tube pumice, microvesicular pumice, DQAs and dense obsidian was analysed using the Cameca SX100 Electron Microprobe at the University of Edinburgh School of Geosciences. DQAs were found to be entirely crystal free, but care was taken to avoid any crystal present in the other pyroclasts. Major elements plus Chlorine and Fluorine were measured against a Lipari obsidian secondary standard. Accelerating voltages and beam sizes were set at 15 keV and 14 μm, respectively, and the beam current was tailored on a per-element basis to minimise diffusive loss (1 nA: Na, Mg, Al, K, Ca and Si; 80 nA: F, Cl, P, S, Ti, Fe and Mn). Analyses with totals <96 wt% were discarded leaving 79 sets of analyses from the range of pyroclasts analysed. The remaining values were normalised to 100% to account for variable secondary hydration. The glass compositions were then compared and the glass densities calculated using the partial densities method^45^.

### X-ray microtomography

A sub-sample of each component class in addition to 3 grades of bulk fine ash was taken and prepared for secondary electron backscatter analysis using the Carl Zeiss SIGMA HD VP Field Emission SEM at the University of Edinburgh School of Geosciences. This provided images for qualitative descriptions of smaller clasts and micron scale surface features. An additional subsample of four FQAs and three DQAs was selected for XCT using the in-house constructed XCT scanner at the University of Edinburgh School of Geosciences. Clasts were measured and the X-ray source—sample distance was adapted for each clast to ensure a whole-clast analysis whilst attaining an optimum resolution. Each sample was rotated around 180° and a series of exposures taken. These images were reconstructed and corrected for beam hardening using Octopus v8.7. For qualitative 3D analysis of the internal structure, these images were loaded into Avizo 9 and a threshold was applied. For quantitative analysis, the image set for each pyroclast was segmented and binarized using the computer-learning based ‘Trainable WEKA Segmentation’ plugin in ImageJ (FIJI)^46^ whereby the user manually trains a classifier to recognise the different phases present (glass vs. vesicle), the classifier then automatically places each pixel into the defined classes. Due to resolution constraints, vesicularities were not directly calculated from the results of the segmentation, instead bulk volumetric measurements of each pyroclast were extracted from the segmented images using the particle analysis function in ImageJ. As these clasts are simple two-phase systems (glass and void space), the bulk density and vesicularity could then be calculated using a mass balance approach by measuring the mass of each clast, and assuming a glass density of 2.47 g cm^−3^ (calculated from the EPMA glass composition data).

### Thermal model

The thermal evolution of FQAs and DQAs during flight through the air was modelled assuming spherically symmetric projectiles with radiative and convective cooling at their outer boundary. For simplicity, and in order to present a maximum cooling rate, conditions were modelled to simulate a ballistic flight path, rather than entrainment in a hot plume or PDC. This provides a temperature profile through the clasts as a function of time. The diffusion of heat through pyroclasts is a function of the thermal conductivity, temperature gradient and the length of the diffusion path. The vesicularity and diameter of these achneliths are coupled and increase through time, thereby reducing thermal conductivity and increasing the length of the diffusion path. However, as we have no independent constraints on the vesiculation and expansion rate, capturing this is beyond the model. Maximum and minimum cooling rate scenarios were modelled instead with a constant diameter and vesicularity throughout flight, thus bracketing the true temperature-time path. A series of pyroclast diameters were modelled with a minimum (not inflated *ϕ* = 0) and maximum (greatest observed inflation *ϕ* = 0.9) vesicularity and the time taken for 10 and 100% of the diameter of each sized pyroclast to cool below *T*_g_ was recorded. As the temporal resolution of the model is a 1 s time-step producing a somewhat stepped relationship, the curves presented in Fig. 5 are best-fit second order polynomials to the model results.

For simplicity, the vesicular achneliths were modelled as an isotropic foam without a skin, and without advective heat loss through gas escape or melt migration to the surface. The thermal properties of this foam were calculated as follows. The thermal conductivity appropriate for this particular peralkaline rhyolite melt (*K*_melt_) was calculated by the method of ref. ^47^, and the thermal conductivity of the gas inside vesicles was neglected allowing the bulk thermal conductivity of the foam (*K*_foam_) to be calculated by the Rayleigh–Maxwell equation:

1 $$K_{\mathrm{foam}} = \frac{{K_{\mathrm{melt}}(1 - {\mathrm{\Phi }})}}{{(1 + {\mathrm{\Phi }})}}{.}$$

The heat capacity of the achneliths (Cp_foam_) was then calculated as the weighted sum of the heat capacity of pantellerite (Cp_melt_) from^48^, and the heat capacity of the vesicle gas (Cp_gas_) (taken as that of steam at 950 K^49^):

2 $${\mathrm{Cp}}_{\mathrm{foam}} = \frac{{{\Phi } {\mathrm{Cp}}_{\mathrm{gas}}} \rho _{\mathrm{gas}} + (1 - {{\Phi }}) {{\mathrm{Cp}}_{\mathrm{melt}}} \rho _{\mathrm{melt}}}{{\rho _{\mathrm{foam}}}}.$$

The thermal diffusivity of the achnelith (*k*_foam_) can then be calculated as^50^:

3 $$k_{\mathrm{foam}} = \frac{{K_{\mathrm{foam}}}}{{\rho _{\mathrm{foam}} {\mathrm{Cp}}_{\mathrm{foam}}}}.$$

Radiative (*F*_r_) and convective (*F*_c_) surface heat flux at the clast surface is calculated as a function of the heat transfer coefficient (*h*_c_), black body emissivity (*γ*) and the temperature at the clast surface (*T*_s_), and the temperature of the surrounding fluid (*T*_∞_) by the following equations:

4 $$F_{\mathrm{c}} = h_{\mathrm{c}}\,(T_{\mathrm{s}} - T_\infty ).$$

5 $$F_{\mathrm{r}} = \gamma \,(T_{\mathrm{s}}^4 - T_\infty ^4).$$

The initial *T*_s_ is 750 °C, the pre-eruptive temperature calculated for peralkaline rhyolites at Aluto using the alkali-feldspar—melt geothermometer^13^. The temperature of the surrounding fluid is 25 °C: standard conditions and a realistic for air temperature at this altitude in Ethiopia. The heat transfer coefficient is a function of the Reynolds (Re) and Nusselt (Nu) numbers:

6 $${\mathrm{Re}} = \frac{{(u\,d\,\rho _{\mathrm{air}})}}{{\mu _{\mathrm{air}}}},$$

7 $$\frac{{h_{\mathrm{c}}\,d}}{{K_{\mathrm{air}}}} = {\mathrm{Nu}} = 2 + \sqrt {0.25 + 3 \times 10^{ - 4} {\mathrm{Re}}^{1.6}},$$

where *u* is the average velocity of the achnelith during cooling, *d* is the diameter of the achnelith, *μ*_air_ is the air viscosity and *K*_air_ is the thermal conductivity of air from^51^. The ‘Eject!’ model of ref. ^39^ was used to calculate *u*, assuming spherical particles with the average diameters and density of pumiceous achneliths, exit velocities of 100 m s^−1^ (realistic for ballistic ejection^41^) and with no zone of reduced drag around the vent.

In the thermal model, the time dependent heat flow equation was discretised as a matrix in spherical coordinates following^52^ and the temporal component was solved using the method of lines^53^. Table 1 shows input parameters used for the thermal model.

**Table 1:** Thermal model parameters

Parameter	Value	Unit
Altitude	1790	m.a.s.l
Density (melt)	2.47^45^	g cm^−3^
Average velocity	30^39^	m s^−1^
Ambient temperature	298.15	K
Initial temperature (melt)	1022.15^13^	K
Density (air)	1.027^54^	kg m^−3^
Viscosity (air)	1.851 × 10^−5^^54^	Pa s
Thermal conductivity (melt)	1.224^47^	W m^−1^ K^−1^
Heat capacity (melt)	1.42^48^	J K^−1^ g^−1^
Density (vesicle gas)	205^55^	g m^−3^
Heat capacity (vesicle gas)	2.343^49^	J K^−1^ g^−1^
Black body emissivity	5.669 × 10^−8^^51^	W m^−2^ K^−4^
Glass transition temperature	673.15^3^	K

## Supplementary information

Supplementary Information

Peer Review File

Description of Additional Supplementary Files

Supplementary Data 1

## Data Availability

Geochemical and field data supporting this work is available within this published article and its supplementary files. 3D models of pumiceous achneliths can be accessed at Clarke et al., Pumiceous Achenliths 3D models, Figshare (https://figshare.com/s/310bc3f3ae076b17fc82). Original reconstructed XCT data are available from the authors on request.

## Code availability

The code for the thermal model is written in Python 3 in Supplementary Code 1 which can be accessed via Github/Zenodo (10.5281/zenodo.2616499).
