# Supplementary material for: Fluidal pyroclasts reveal the intensity of peralkaline rhyolite pumice cone eruptions
Source: Nat Commun. 2019 May 1;10:2010. doi: 10.1038/s41467-019-09947-8 (PMC6494994; doi:10.1038/s41467-019-09947-8)
Supplement: Supplementary file 1 — Supplementary Information [file 41467_2019_9947_MOESM1_ESM.pdf]

# Fluidal pyroclasts reveal the intensity of peralkaline rhyolite pumice cone eruptions

Clarke et al.

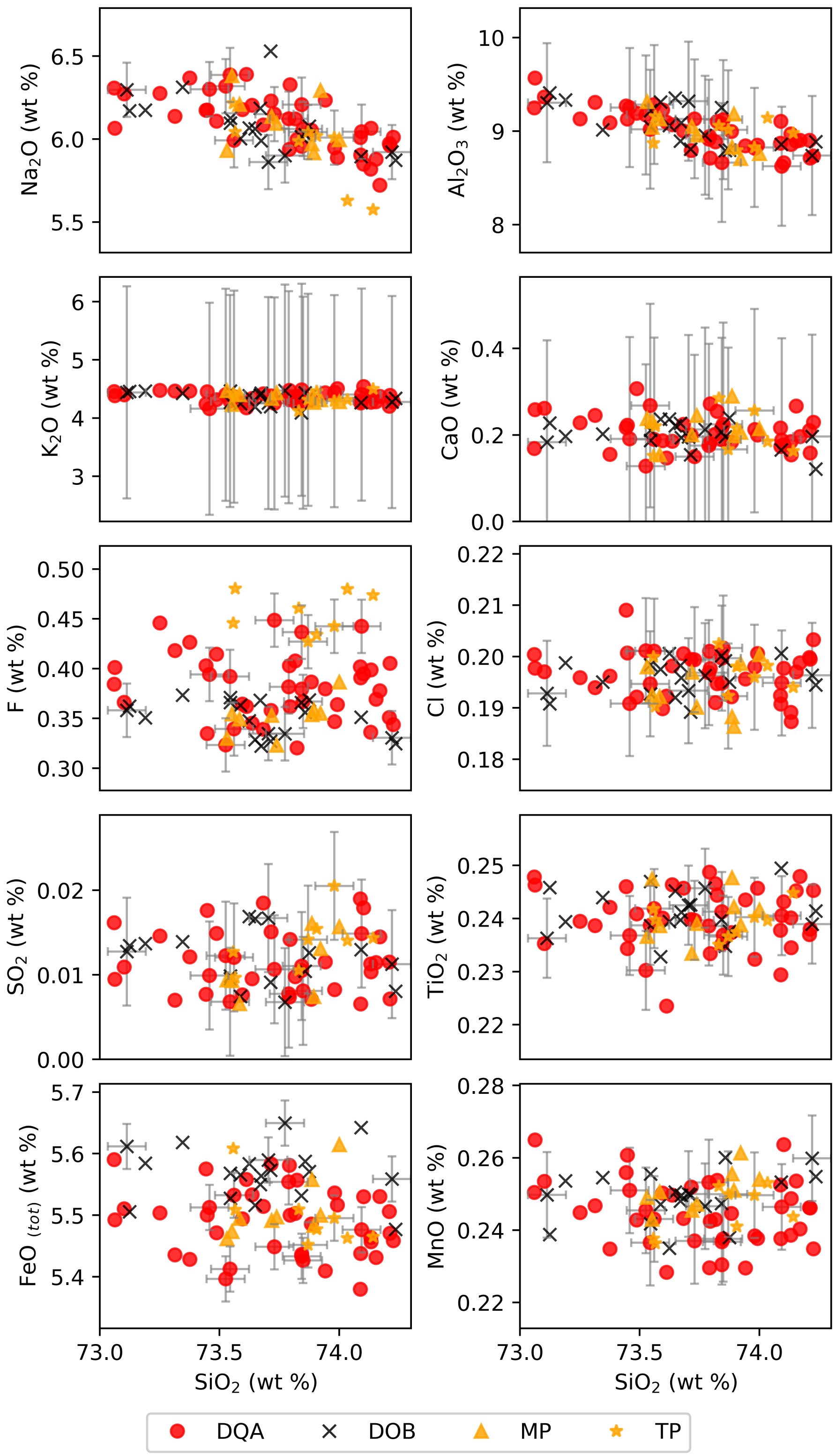


Supplementary Figure 1: Harker plots comparing deposit components: **DQA** deposit quenched achnelith, **DOB** dense obsidian bread crust bomb, **MP** microvesicular pumice and **TP** tube pumice. Error bars represent ± 1 standard deviation of the standard data for each element.

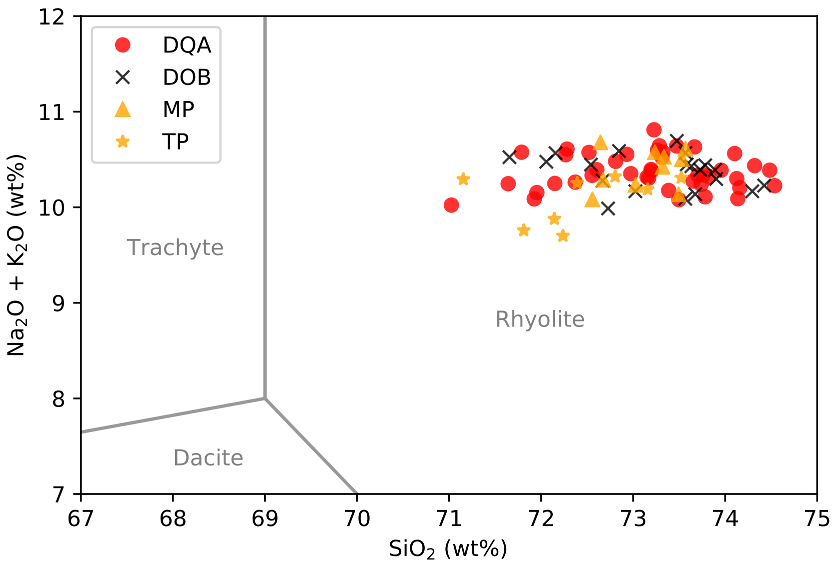


Supplementary Figure 2: Total alkali – silica classification of deposit components: **DQA** deposit quenched achnelith, **DOB** dense obsidian bread crust bomb, **MP** microvesicular pumice and **TP** tube pumice.

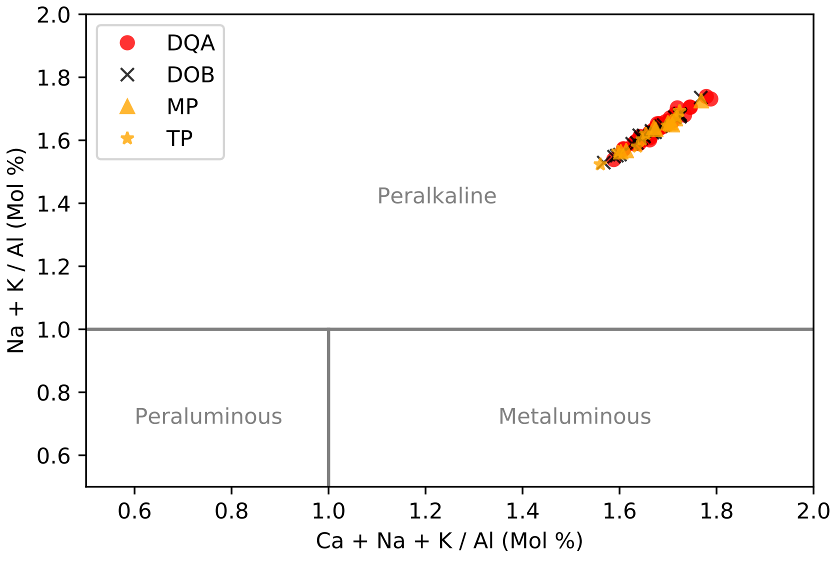


Supplementary Figure 3: Peralkaline classification of deposit components: **DQA** deposit quenched achnelith, **DOB** dense obsidian bread crust bomb, **MP** microvesicular pumice and **TP** tube pumice.

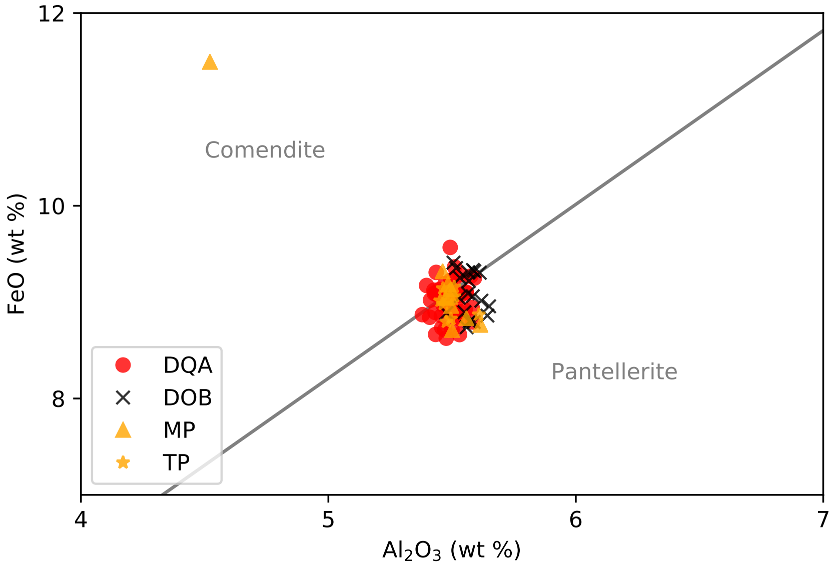


Supplementary Figure 4: Sub-divided peralkaline classification of deposit components: **DQA** deposit quenched achnelith, **DOB** dense obsidian bread crust bomb, **MP** microvesicular pumice and **TP** tube pumice.
