# Supplementary material for: Fluidal pyroclasts reveal the intensity of peralkaline rhyolite pumice cone eruptions
Source: Nat Commun. 2019 May 1;10:2010. doi: 10.1038/s41467-019-09947-8 (PMC6494994; doi:10.1038/s41467-019-09947-8)
Supplement: Supplementary file 3 — Description of Additional Supplementary Files [file 41467_2019_9947_MOESM3_ESM.pdf]

Description of Additional Supplementary Files

File Name: Supplementary Data 1
Description: Geochemical dataset including the major-element, F and Cl analyses of glass from different pyroclast types sourced from the north sample site. Standard analyses of Lipari Obsidian are also provided. The data is derived from Electron Probe Micro Analysis (EPMA) conducted at the University of Edinburgh School of Geosciences. Element-wise EPMA conditions are provided within the dataset.'
