# Supplementary material for: Fluidal pyroclasts reveal the intensity of peralkaline rhyolite pumice cone eruptions
Source: Nat Commun. 2019 May 1;10:2010. doi: 10.1038/s41467-019-09947-8 (PMC6494994; doi:10.1038/s41467-019-09947-8)
Supplement: Supplementary file 2 — Peer Review File [file 41467_2019_9947_MOESM2_ESM.pdf]

Reviewers' comments:

Reviewer #1 (Remarks to the Author):

The is a well-founded and highly relevant study which will be a vaulable addition to the physical volcanology literature.

My comments are minor.

line 35 , refernce 6 seems slightly out of place here.

1line 58 "inflated achneliths" . This term is a genetic one. Perhaps a generic non-interpretational term would be superior. Are not basaltic achneliths also expanded?

line 66 do these samples contain fresh/pristine glass?

line 105 have you added the fluorine to water to determine the viscosity?

line 167 "inertial fragmentation" appears to date from a time before the application of physics to the fragmentation problem and I suggest that it may be a dated term. As to whether the fragmentation is ductile or brittle, those are no clearly defined in the present paper. All fragmentation may involve a brittle element.

line 177 a reference to Dingwell and Webb, 1989 after the Maxwell equation would be appropriate. After all you have cited Thomas and Sparks for the thermal conductivity of air!

line 184 reference(s ?) to the data that demonstrate this equivalence (basalt and pantellerite plus water) would be appropriate here.

line 195 I do not think that you can distinguish brittle from ductile. I believe you can merely distinguish those samples containing renmant brittle morphological elements and those which do not. But the preservation of brittle textures is something decided by the flight fall and welding thermal regime alone.

line 206 I do not think it challenging. The decompressive stresses have to be applied (accelerated) to the magma sufficiently quickly. More is not needed.

line 256 the distinction between breadcrustring or not is likely to be a delicate and gradual transition in time-temperature-rheology contrast fron interior to exterior where in the case of breadcrusting critical stresses have been overcome. It is a gradual transition and not a hard boundary between two different mechanisms

Reviewer #2 (Remarks to the Author):

This is a well-structured and detailed study of peralkaline explosive eruptions.

There are a few instance of unnecessary, and somewhat jarring hyperbolae e.g lines 48-49 which detract from the reading. Removing these would shorten the text without adversely affecting the science and improve readability.

An additional figure, or an amendment of an existing one to better show the bubble scale textures inside the samples would be extremely beneficial. Figure 2 is a nice example of the 3D, but the jump from that to Fig 4. Is too extreme and does not give the reader a clear understanding of the textures as described in the text.

Please change all instances of µ-CT to XCT or XMT. µCT does not indicate what modality of tomographic imaging you are using (x-ray, neutron, diffraction, fluorescence, XANES etc) and all X-ray tomography is essentially micro-tomography. There is an ongoing effort to standardise the terminology to XCT or XMT (sXMT/sXCT for synchrotron based image acquisition).

Is there an inherited component of deformation/inflation in flight in any of the clasts that then see further inflation in deposit? Ridges on DQA?

Is there a role of strain rate on the onset of brittle behaviour? The initial fragmentation of the melt is, at least in part, likely to be strain rate controlled. Reiterating a little of your discussion at the top of Pg 12 into your conclusions on pg 18 would be good – pulling in the evidence for heterogeneity in the conduit and fragmentation. What is potential role of that heterogeneity in the melt being supplied and the supply rates/fragmentation mechanisms on the eruption column and hazard assessment?

A lot of references are in line, making it harder to follow the text.
Two examples at Ln 150: Change to "…400C (using Di Genova t al XXXX)." and at Ln 170-171 change to "…by surface tension, making them 'achneliths' (Walker and Croasdale), a form…" but similar usage elsewhere should be removed wherever possible, in text and figure captions.

Ln 140: Replace Accidental with Occasional or rare.
Ln 143-156 Please clarify how clast diameter was derived. Is this the pre inflated diameter for the DAQ. If not why cannot an assessment of the pre inflation size be made? Cooling is occurring during inflation in these samples?
Ln 230 remove typically – already have "more often" and don't need both
Ln 257 mis-formatted reference? (7)

Please strengthern the elements of the discussion on the potential impact of the study in terms of hazard etc. A lot is made of this at the start and by the end it feels little lacking.

Ln 360 A little more detail on how the WEKA segmentation was performed would be useful to the non-CT specialists among ther readership, both in the general terms and the specifics. Your reader should be able to reproduce your data from your manuscript and your raw data file. I know there is a degree on manual decision making in the process, but still, more information is required. How did you test how "well" your segmentation was working? What are the errors and uncertainty in your CT derived values? Did you try and use greyscale values including partial volume values as more robust volume fraction porosity and mass calculation? You can assume thick films are 100% solid and bubbles centres are 100% air, with linear rage of partial volume between those values.

Dear Reviewers,

Thank you for your constructive comments on our manuscript. We have modified the manuscript to address your concerns and believe this has significantly improved the manuscript. In particular, we have emphasised the implications for hazards at peralkaline rhyolite volcanoes and explored in greater detail the nature of fragmentation during these eruptions. Edited sections are highlighted in the manuscript, and we respond to your comments individually:

Reviewer 1

1. **line 35 , reference 6 seems slightly out of place here.**
   We have removed this reference
2. **line 58 "inflated achneliths". This term is a genetic one. Perhaps a generic non-interpretational term would be superior. Are not basaltic achneliths also expanded?**
   We have changed the name to *'pumiceous achneliths'* throughout the manuscript, which removes any genetic implications, but reflects the high vesicularity and bubble number density of the pyroclasts compared to any more 'typical' basaltic achneliths the authors have seen
3. **line 66 do these samples contain fresh/pristine glass?**
   Added sentence (line 112) to clarify:
   'In thin section, the glasses are isotropic with no apparent alteration, suggesting they are relatively fresh and similar to their original form.'
4. **line 105 have you added the fluorine to water to determine the viscosity?**
   We have not added Fluorine to water. In the Di Genova model (which we use to model the viscosity), the effect of F is not explicitly considered. However, as they used natural obsidian flow derived samples, F was present in the glass and will have influenced their measured viscosities, and so is implicitly accounted for in the model. If the F contents of Aluto glasses are similar those measured by Di Genova, not adding F to water would be appropriate (so not to take F into account twice). Unfortunately, Di Genova et al. don't report the F concentration in their samples. However, Gioncada and Landi (2010) measured the F content of olivine and feldspar MIs from pumices produced during the same eruption and find F concentrations of 2400 to 6000 ppm (mean 3900ppm). This is very similar to Aluto (mean F=3700 ppm) and so we consider the model to have already taken F into account without having to add it to water. We have added the following sentences the manuscript as clarification (line 184 to 189):
   'It should be noted that F has a network modifying influence on the viscosity of silicate melts (Dingwell, 1985) of the same magnitude as water on a wt % basis (Giordano 2004), and the peralkaline rhyolite viscosity model (Di Genova, 2013) does not explicitly take F into account in model coefficients. However, F concentrations in the samples used to calibrate the model (pumice from the same eruption: mean 3900 ppm F (Gioncada 2010)) are very similar to those measured in glasses at Aluto (mean 3700 ppm F). The model is therefore considered to implicitly take the influence of F into account for the Aluto samples.'
5. **line 167 "inertial fragmentation" appears to date from a time before the application of physics to the fragmentation problem and I suggest that it may be a dated term. As to whether the fragmentation is ductile or brittle, those are no clearly defined in the present paper. All fragmentation may involve a brittle element.**
   We have rephrased the sentence to replace *'inertial fragmentation'*, with *'ambiguous, non-*

brittle' fragmentation (line 173), at the suggestion of the reviewer (Pers. Comm). The nature of fragmentation is further explored below in response to reviewer 1 comments 8 and 9

6. **line 177 a reference to Dingwell and Webb, 1989 after the Maxwell equation would be appropriate. After all you have cited Thomas and Sparks for the thermal conductivity of air!**
   We have added this reference (line 182)
7. **line 184 reference(s ?) to the data that demonstrate this equivalence (basalt and pantellerite plus water) would be appropriate here.**
   We have added references citing the origin of the chemical standard data and the viscosity calculator used to derive these viscosities (line 195 & figure 6)
8. **line 195 I do not think that you can distinguish brittle from ductile. I believe you can merely distinguish those samples containing remnant brittle morphological elements and those which do not. But the preservation of brittle textures is something decided by the flight fall and welding thermal regime alone.**
   We have rephrased the paragraph beginning line 202 and added points to clarify. In particular, we recognise that the lack of remnant brittle features (as observed in many basaltic achneliths) cannot exclude a brittle origin (line 213), but instead that the presence of remnant brittle features on these achneliths confirms their brittle origin
9. **line 206 I do not think it challenging. The decompressive stresses have to be applied (accelerated) to the magma sufficiently quickly. More is not needed.**
   In the paragraph beginning line 216, we have extended the discussion on the role of strain-rate on the fragmentation of these low viscosity melts. We also argue that strain localisation is both necessary, and independently evidenced by the variety of pumice textures, in order to brittlely fragment these melts under geologically reasonable eruption conditions
10. **line 256 the distinction between breadcrusting or not is likely to be a delicate and gradual transition in time-temperature-rheology contrast from interior to exterior where in the case of breadcrusting critical stresses have been overcome. It is a gradual transition and not a hard boundary between two different mechanisms**
    We have rephrased the paragraph beginning line 231 to indicate that the regimes 1 and 2 are end members and are a result of a fine balance of various rates/timescales. However, as critical stresses must be overcome in order to develop breadcrust textures, there must be distinct regimes

Reviewer 2

1. **There are a few instances of unnecessary, and somewhat jarring hyperbolae e.g lines 48-49 which detract from the reading. Removing these would shorten the text without adversely affecting the science and improve readability.**
   We have rephrased lines 44 to 46 to address this whilst retaining the motivation for studying EAR volcanoes in particular:
   'The East African Rift (EAR) contains one of the greatest densities of silicic peralkaline volcanoes on Earth, and furthermore, Ethiopia is regarded as the fifth most volcanically threatened country (Loughlin 2015); with 10 million people living within 30 km of a volcano (Aspinall, 2011).'

2. **An additional figure, or an amendment of an existing one to better show the bubble scale textures inside the samples would be extremely beneficial. Figure 2 is a nice example of the 3D, but the jump from that to Fig 4. Is too extreme and does not give the reader a clear understanding of the textures as described in the text.**
   We have added three additional images to Figure 2 which show more clearly the cross-sectional structure of the achneliths. We combine reconstructed and binarized XCT slices with SEM backscatter images of the achneliths which is informative for both the structure of the achneliths, but also indicative of the quality of the binarization and the resolution of the original XCT

3. **Please change all instances of µ-CT to XCT or XMT. µCT does not indicate what modality of tomographic imaging you are using (x-ray, neutron, diffraction, fluorescence, XANES etc) and all X-ray tomography is essentially micro-tomography. There is an ongoing effort to standardise the terminology to XCT or XMT (sXMT/sXCT for synchrotron based image acquisition).**
   This has been changed to XCT in all instances

4. **Is there an inherited component of deformation/inflation in flight in any of the clasts that then see further inflation in deposit? Ridges on DQA?**
   The ridges on the DQA are pointed out in Figure 2, but we interpret this to be an inherited component from fragmentation rather than any in-flight processes. We do see transitional clast morphologies (ie. Spatter-form droplets (Fig.2 C) which are clasts that were clearly droplets (thus deformed in-flight) but have deformed after deposition. We have clarified this on line 207:
   '…point-and-ridge structures on the surface of many achneliths (both FQAs and DQAs)…'

   and added a further sentence about transitional 'spatter-form' achneliths on line 285:
   '…as well as transitional `spatter-form' clast morphologies (Figure 2c) which were clearly small and droplet shaped during flight but deformed on impact.'

5. **Is there a role of strain rate on the onset of brittle behaviour? The initial fragmentation of the melt is, at least in part, likely to be strain rate controlled. Reiterating a little of your discussion at the top of Pg 12 into your conclusions on pg 18 would be good – pulling in the evidence for heterogeneity in the conduit and fragmentation. What is potential role of that heterogeneity in the melt being supplied and the supply rates/fragmentation mechanisms on the eruption column and hazard assessment?**
   This has been addressed in the same response to reviewer 1's comments. In the concluding discussion, we now also suggest that the presence of strain localisation, and heterogenous magma viscosities across the conduit might impart temporal and spatial changes in style and thus column instabilities which can promote the generation of pyroclastic density currents

6. **A lot of references are in line, making it harder to follow the text.**
   Two examples at Ln 150: Change to "…400C (using Di Genova t al XXXX)." and at Ln 170-171 change to "…by surface tension, making them 'achneliths' (Walker and Croasdale), a form…" but similar usage elsewhere should be removed wherever possible, in text and figure captions.
   We have changed inline references where it made sense to do so

7. **Ln 140: Replace Accidental with Occasional or rare.**
   (line 140) We have replaced *'accidental'* with *'conduit-derived, accessory lithic'* to reduce ambiguity

8. **Ln 143-156 Please clarify how clast diameter was derived. Is this the pre inflated diameter for the DAQ. If not why cannot an assessment of the pre inflation size be made? Cooling is occurring during inflation in these samples?**
   We have recalculated the original sizes of the achneliths (assuming an original 0 porosity, and a final 0.9 porosity) and have now provided histograms in Figure 5. This now displays both the original and inflated size distributions of the pyroclasts. The impact of a changing diameter through flight on the cooling times is reflected by coloured ranges for the mean FQA and DQA. The methods for calculating clast diameter are provided in lines 363 to 365.

9. **Ln 230 remove typically – already have "more often" and don't need both**
   This sentence has been re-written (line 248):
   'This explains why larger bread crust textures develop more often in the larger DQAs than in the smaller FQAs.'

10. **Ln 257 mis-formatted reference? (7)**
    Fixed, refers to a figure

11. **Please strengthen the elements of the discussion on the potential impact of the study in terms of hazard etc. A lot is made of this at the start and by the end it feels little lacking.**
    Hazard assessment at any volcano needs to be founded on a sound conceptual model for the eruptive behaviour of the system in question. The shift in understanding of pumice cone forming eruptions provided by this work: from strombolian (where eruptive hazards are limited to proximal locations), to eruptions that can generate (unstable) convective columns (typically resulting in widespread tephra fall and PDCs), is an important step in understanding the scope and range of hazards at these volcanoes. It is this, rather than precise, quantitative information on aspects such as plume height, eruptive volume or pyroclastic density current runout, that can be provided through this work. However, we have extended the discussion (from line 321) to emphasise these points, and provide comparisons with recent, potentially somewhat similar eruptions, which should hopefully better communicate the important hazard implications of this research. We have also included the influence of likely impact of conduit heterogeneities on the stability of the tephra columns produced.

12. **Ln 360 A little more detail on how the WEKA segmentation was performed would be useful to the non-CT specialists among ther readership, both in the general terms and the specifics. Your reader should be able to reproduce your data from your manuscript and your raw data file. I know there is a degree on manual decision making in the process, but still, more information is required. How did you test how "well" your segmentation was working? What are the errors and uncertainty in your CT derived values? Did you try and use greyscale values including partial volume values as more robust volume fraction porosity and mass calculation? You can assume thick films are 100% solid and bubbles centres are 100% air, with linear rage of partial volume between those values.**
    We have added a brief description on how WEKA segmentation is performed (line 389). In terms of assessing the segmentation quality: We visually compared the binarized output to the reconstructed CT slice in addition to the viewing the certainty estimates provided by the WEKA segmentation. We also compared the scale of visible structures apparent in the SEM

backscatter to those in the reconstruction and the segmentation. We found that the XCT resolution wasn't sufficient to image the smallest bubbles we could see in the backscatter, and that the segmentation struggled with thin bubble walls. Qualitatively therefore, the segmentation wasn't good for assessing bubble size, shape and distribution (despite efforts improve segmentation and watershed). However, the exterior skin of the clasts were always well segmented, with high certainty on the output from the WEKA segmentation. For that reason, we used a mass balance approach to estimate the porosity of the clasts, where the only important quantitative information from the XCT was the bulk clast volume (which we could reliably ascertain from the exterior skin). This approach is described in the methods (line 395). The greatest value of the XCT in this instance was for the development of the 3D images to assess overall structure, and so we deemed a simple thresholding approach to be adequate. In summary: our XCT data was not sufficient for detailed qualitative analysis of the vesicles. However, it was adequate for qualitative analysis of the overall clast structure and for bulk volume measurement. A more detailed exploration of segmentation quality would be necessary for a detailed study of vesicle structure, but in this instance we feel is superfluous, and might unnecessarily extend the paper

We hope that we have adequately addressed your concerns; we do feel we have a stronger manuscript as a result.

Yours Sincerely,

Ben Clarke

REVIEWERS' COMMENTS:

Reviewer #1 (Remarks to the Author):

I would like to thank the authors for serious consideration of the previous comments of the referees. I feel that many important points are better clarified now and the readability of the paper is improved for broader readership of Nature Communications. I still feel that the entropic contributions must be better accounted for in forthcoming publications. For the time being I am sufficiently convinced by the authors. The observed rich library of superstructures observed in a relatively simple model is an interesting result, which can stimulate further theoretical and experimental studies. I am therefore pleased to recommend the MS for publication in Nature Communications. Andrei Petukhov

Reviewer #2 (Remarks to the Author):

The reply is convincing. I recommend publication.

Reviewer #3 (Remarks to the Author):

Second report for “Using symmetry to elucidate the importance of stoichiometry in colloidal crystal assembly” by Mahynski et al.

The authors have done a reasonable job of addressing the points raised in the first round of reveiwing, and indeed the manuscript is much shorter and more accessible. I am now happy to recommend the manuscript for publication.
